# Reading the Mind through the Nose: Mentalizing Skills Predict Olfactory Performance

**DOI:** 10.3390/brainsci12050644

**Published:** 2022-05-13

**Authors:** Katrin T. Lübke, Tobias C. Blum, Bettina M. Pause

**Affiliations:** Department of Experimental Psychology, Heinrich-Heine-University Düsseldorf, D-40225 Düsseldorf, Germany; tobias.blum@hhu.de (T.C.B.); bettina.pause@hhu.de (B.M.P.)

**Keywords:** olfaction, olfactory discrimination, social skills, empathy, theory of mind, mentalizing, gender

## Abstract

A growing body of literature suggests a close link between olfaction and social expertise. The current study examines whether mentalizing skills are related to olfactory discrimination performance. In order to assess their mentalizing ability, 21 women and 20 men completed the “Reading the Mind in the Eyes” test (RMET). Here, the participants have to infer other persons’ mental state from photographs of eye regions. Odor discrimination was assessed using the “Düsseldorf Odour Discrimination Test” (DODT). The DODT consists of 15 items, each containing mixtures of four monomolecular substances. Within each item, two bottles contain the same mixing ratio, while the third contains the reversed mixing ratio. The participants had to identify the deviating odor. Women’s expertise in mentalizing (RMET score) is strongly related to olfactory discrimination performance (DODT score): The better women are in mentalizing, the better their performance in olfactory discrimination (rho = 0.572, *p* = 0.042, Bonferroni-corrected). Men’s mentalizing capability was unrelated to olfactory discrimination (rho = −0.117, *p* > 0.999, Bonferroni-corrected). The current results suggest that the social skill of mentalizing might underly the link between olfaction and social integration, at least in women, and are discussed with regard to the social nature of human olfaction.

## 1. Introduction

In recent years, results accumulate indicating that the human sense of smell is closely related to aspects of social life. Typically, higher social functioning is associated with better olfactory performance. Research has shown a positive relationship between olfactory function and the size of one’s social network [1]. Moreover, within a large representative sample of older adults, both the size and the quality of one’s social network (number of close friends, number of family members one feels close to, and frequency of socializing) showed a positive correlation with olfactory performance [2]. Smell loss, on the other hand, is associated with social insecurity and impairment across different types of social relationships (for a recent review, see [3]).

The question remains, what are the psychological underpinnings relating social integration to olfactory performance. On one hand, there are social skills such as empathy, which can be defined as “emotional and mental sensitivity to another’s state” [4]. Empathy is considered to be crucial in almost any social interaction, since it aids in the understanding and prediction of others’ behavior [5] and is—comprehensibly—related to social network size (e.g., see [6,7]). Intriguingly, first studies show that the more empathetic individuals report to be, the better their olfactory performance is [8,9]. Moreover, individuals who self-describe as exceptionally social skilled show enhanced activation within the mirror neuron system upon exposure to social odors [10], supporting a link of olfaction and social skills to cognitive empathy. On the other hand, there are affective states related to social network size, such as feelings of loneliness and depression when social integration is low [11], or feelings of happiness when social integration is high [12,13]. Affective states have been shown to affect olfaction [14,15], and especially sadness and depression have been linked to olfactory deficits (e.g., see [16]). In addition, and independent of the experience of sadness and depression, reports of loneliness show a negative relationship to olfactory function [17].

Gender affects social and empathic skills, olfactory performance, and their mutual interrelations. In olfaction research, it is a common finding that women outperform men [18]. The pattern is similar in social and empathy-related research [19,20], especially when social cues are subtle [21]. Finally, findings of correlations between olfaction and social functioning are especially pronounced in women [2,22]. Women, but not men, show better olfactory abilities the richer they describe their social life (number of friends, closeness to relatives, frequency of socializing, see [2]). Moreover, in women, but not in men, attachment insecurity is negatively correlated to olfactory abilities [22].

The aim of the current study is to uncover the psychological aspects linking social to olfactory competencies. Here, the concept of empathy is specified, focusing on the cognitive component of empathy, including perspective taking and understanding someone’s emotions. Furthermore, it is intended to assess empathy performance instead of self-descriptions, as the latter are especially prone to be distorted by social desirability. In detail, the current study examines whether the individual expertise in mentalizing, i.e., the ability to form accurate mental representations of another person’s (and even one’s own) mental state, is related to olfactory discrimination performance in men and women. According to Baron-Cohen et al. [23], mentalizing, or “theory of mind”, is “the main way in which we make sense of or predict another person’s behavior” (p. 241). Within the current study, mentalizing is assessed by means of the “Reading the Mind in the Eyes” test (RMET [23]). Moreover, measures of loneliness and happiness are taken since both feelings are intimately linked to social integration. Olfactory discrimination, as assessed by means of the “Düsseldorf Odour Discrimination Test” (DODT [24]), was chosen since the ability to detect quality differences between odors is probably the most innate characteristic of the olfactory system, and is not prone to effects of verbal skills (such as identification) or the sensitivity for a specific odor (such as threshold measurements; for discussion, see [24]). As an additional measure of olfactory performance, the “Brief Smell Identification Test” (BSIT; Sensonics Inc., Haddon Heights, NJ, USA) was introduced. However, in comparison to odor discrimination, odor identification performance is rather prone to be affected by cultural odor preferences, verbal skills and cognitive competencies.

It is expected that both mentalizing and happiness are positively correlated to olfactory discrimination performance, while loneliness should show a negative relationship with olfactory discrimination. As these relationships are expected to be evident in women, but not in men, men and women are analyzed separately. Since there is tentative evidence linking sex hormones to both olfaction and empathy (for discussion, see [25]), the level of free estradiol and testosterone was measured exploratorily.

## 2. Materials and Methods

### 2.1. Participants

The female sample consisted of 21 women with a mean age of 25 years (SD = 5 years, range = 19–38 years), and the male sample consisted of 20 men with a mean age of 24 years (SD = 3 years, range = 20–29 years). Men and women did not differ in age (*p* = 0.457). According to self-report, none of the male and female participants suffered from any neurological, psychiatric, endocrine, or immunological diseases, or acute or chronic diseases of the respiratory tract. All were non-smokers, reported not using any drugs and had normal or corrected vision. Female participants were not using any hormonal contraception and reported having a regular menstrual cycle. In order to minimize menstrual cycle effects on olfactory performance [26], they were required to attend the study session during the second half of their menstrual cycle. Further, none of the participants showed a social desirability bias (assessed with the Social Desirability Scale 17, (SDS) [27], German adaption by Stöber [28]). Male and female participants gave written informed consent and were compensated either financially or with course credits. The study was conducted in accordance with the Declaration of Helsinki and approved by the ethics committee of the Faculty of Mathematics and Natural Sciences of the Heinrich-Heine-University Düsseldorf (Düsseldorf, Germany).

### 2.2. Materials

#### 2.2.1. Mentalizing

Mentalizing performance was assessed using the German translation [29] of the RMET [23]. This task consisted of 36 photographs of the eye region expressing complex emotional states (e.g., playful or skeptical). In a computer-assisted multiple-choice format, four adjectives were presented with each photograph (e.g., indifferent, embarrassed, skeptical, and dispirited). The participants had to select the adjective which best described what the depicted individual was thinking, feeling or expressing. Each picture was presented for as long as the participant needed to select an answer. Accuracy performance was calculated as the number of correctly identified expressions (range = 0–36). Presentation 18.3 (Neurobehavioral Systems Inc., Albany, CA, USA) was used to control the RMET procedure.

#### 2.2.2. Happiness and Loneliness

Happiness was assessed by asking the participants to indicate their current level of happiness on a scale ranging from 0 to 10, with 0 being unhappiest and 10 being happiest [30]. Further, the German version of the UCLA Loneliness Scale was used to assess the level of individual loneliness, ranging from 1 (least lonely) to 5 (most lonely, [31,32]).

#### 2.2.3. Olfactory Performance

All participants were tested individually in an air-conditioned room (temperature: 19–21 °C). Odor discrimination performance was assessed via the DODT [24]. In each of the 15 items, three bottles were presented, each containing a mixture of the same four monomolecular odors (out of a total of six odors used throughout the test, each diluted in diethyl-phthalate: coumarin 1:5, decanoic acid 1:40, eugenol 1:40, geraniol 1:40, phenethyl alcohol 1:10 and vanillin 1:10). The mixing ratio of the deviant bottle was complementary to that of the other two bottles (which were identical to one another). The participants were allowed to take one single natural sniff of each bottle within a given test item, separated by at least 6 s, and then required to choose the deviant bottle (forced choice). According to the number of test-items solved, the discrimination score varies between 0 (worst performance) and 15 (best performance).

The ability to identify odors was determined using the BSIT (Sensonics Inc., Haddon Heights, NJ, USA), a 12-item scratch and sniff forced choice test with four descriptors per item [33]. Scores vary between 0 (worst performance) and 12 (best performance).

#### 2.2.4. Saliva Sampling and Hormone Detection

Participants refrained from meals and any beverages except for water at least 60 min prior to the beginning of the session. The sessions were conducted in the afternoon, six to nine hours after awakening. In order to reduce effects related to the periodic secretion patterns of steroid hormones, three saliva samples were collected over the course of 30 min. Passive drooling devices (Salicaps, IBL International GmbH, Hamburg, Germany) were used for sampling, and samples were frozen at −20 °C. Mixed aliquots of each participant’s samples were analyzed for levels of free testosterone and estradiol. Analyses were conducted by means of commercially available enzyme-linked immunosorbent assays with chemiluminescence detection (IBL International GmbH, Hamburg, Germany).

### 2.3. Data Analysis

Within each gender (*n* = 21 women, *n* = 20 men), correlations of mentalizing ability, happiness, and loneliness with olfactory performance (DODT, BSIT) were analyzed. Moreover, the relationships of steroid hormone levels (estradiol, testosterone) and olfactory performance were examined. One woman’s saliva samples could not be analyzed due to handling errors, and one man’s saliva samples appeared contaminated with blood, thus the respective analyses are based on *n* = 20 women and *n* = 19 men. Since happiness ratings, olfactory identification scores (BSIT) and the testosterone level were not normally distributed within one or both genders (Kolmogorov–Smirnoff test, all *p*_s_ ≤ 0.015), non-parametric Spearman correlations on rank data were calculated. In order to reduce the family-wise error rate, results were subjected to Bonferroni corrections within each gender, and separately for the analyses of the relationships between social characteristics (mentalizing, happiness, and loneliness) and olfactory performance (DODT, BSIT) on one hand, and the exploratory analyses of the relationships between sex hormone levels and olfactory performance (DODT, BSIT) on the other hand. Fisher’s z-transformation was used to compare the strength of given significant correlations of two parameters between men and women [34]. Confidence intervals are reported according to Fieller et al. [35]. The alpha level was set at α = 0.05, and all analyses were conducted using IBM SPSS Statistics 28. G*power was used for power analyses [36].

## 3. Results

Table 1 gives a general impression on the overall performance, ratings and the levels of estradiol and testosterone in women and men.

### 3.1. Female Sample

**Figure 1:**
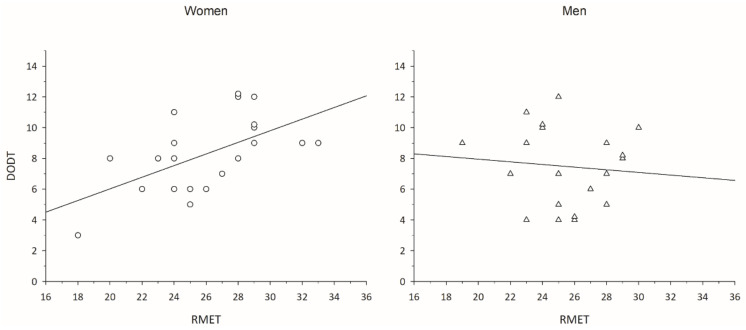
Women’s (left column, dots) and men’s (right column, triangles) data distribution of mentalizing expertise (“Reading The Mind In The Eyes” test; RMET) by olfactory discrimination performance (“Düsseldorf Odour Discrimination Test”; DODT). For better visualization, raw instead of rank data are displayed, and the RMET range is reduced (total range: 0–36).

Women’s expertise in mentalizing (RMET) shows a strong positive correlation with olfactory discrimination performance (DODT; RMET x DODT: rho = 0.572, *p* = 0.042, Bonferroni-corrected, 95% CI [0.173, 0.810], power = 0.81, see Figure 1). The only other noticeable correlation emerged between happiness ratings and olfactory discrimination, which did, however, not survive Bonferroni corrections (see Table 2). Still, in order to control for any effect of happiness on the relationship between olfactory discrimination and mentalizing, a non-parametric partial correlation was conducted [37]: When controlling for happiness, the relationship between the RMET and DODT scores remains significant (partial rho = 0.463, *p* = 0.040), showing that women indeed perform better in olfactory discrimination the better their mentalizing expertise is, irrespective of their current happiness. No further relationships with olfactory discrimination emerged, and olfactory identification (BSIT) was totally unrelated to any other scale or performance (see Table 2). Mentalizing, happiness and loneliness were not significantly interrelated (uncorrected *p*_s_ ≥ 0.056), nor were olfactory discrimination and identification (uncorrected *p* = 0.496). Estradiol and testosterone showed a positive correlation (rho = 0.537, *p* = 0.015).

### 3.2. Male Sample

Neither measure of olfactory performance was related to any rating or performance in men (see Table 3). Mentalizing, happiness and loneliness were not significantly interrelated (uncorrected *p*_s_ ≥ 0.136), nor were olfactory discrimination and identification (uncorrected *p* = 0.458) or estradiol and testosterone, respectively (uncorrected *p* = 0.611). Importantly, men’s mentalizing capability was unrelated to olfactory discrimination (rho = −0.117, *p* > 0.999, Bonferroni-corrected, 95% CI [−0.542, 0.356], power = 0.08, see Figure 1), in contrast to women. 

### 3.3. Female vs. Male Sample

Direct comparison shows that the correlation between mentalizing and olfactory discrimination is significantly higher in women as compared to men (z = 2.206, *p* = 0.027).

## 4. Discussion

The current study shows a substantial positive relationship between mentalizing skills and olfactory discrimination performance in women: the more accurate women are in forming mental representations of another person’s psychological state, the better their ability to detect subtle quality differences between odors. Given that empathy is fundamental for social interaction, it might thus be one of the key psychological skills linking social to olfactory competencies.

The close relationship between empathic skills and olfactory performance is well in line with earlier findings [8,9]. The current research, however, expands these findings beyond the level of mere self-reports of empathy, as it focuses on empathy-related performance. Olfaction and empathy show overlapping neural substrates, as parts of the primary (amygdala) and the secondary olfactory cortex (orbitofrontal cortex, thalamus) are also involved in empathic processes and mentalizing [38,39,40]. As highlighted within the introduction, empathy and the overlapping mentalizing are essential skills for successful social interaction. The neuroanatomical overlap between olfaction and empathy, together with the current results showing a substantial functional correlation, suggest that olfactory and social skills are linked intimately. Indeed, several lines of research strongly support this notion, i.e., findings of social integration related to olfaction [1,2,17], the involvement of olfaction in the human chemonsensory communication of social information (for an overview see [41]), the genetic homophily between the olfactory genome of friends [42], or the discussion of “higher olfactory functions” having been engaged in the evolution of social behavior in modern humans [43].

Intriguingly, within the current study, mentalizing skills are the only “social parameter” which is correlated to olfactory performance. Neither self-rated happiness nor reports of loneliness show any substantial relationship to olfactory performance. Given the fact that mentalizing and the overlapping cognitive empathy are fundamental skills in successful social interaction [5], it is highly conceivable that they affect the degree of social integration and the resulting level of happiness. The current results thus suggest that relationships between loneliness and olfaction, as reported earlier in [17], might be secondary to the link between olfactory and social skills.

In contrast to earlier studies [8,9], the current results specifically relate empathic skills to olfactory discrimination, and not to olfactory identification. So far, olfactory discrimination performance has rather been suggested to be related to autistic traits within the general population [44]. Within the current sample of healthy young adults, olfactory identification performance as measured via the BSIT only showed marginal variance, indicating that the BSIT did not detect finer differences in olfactory performance. In contrast, the DODT was designed to be highly sensitive to detect small differences in olfactory performance specifically between healthy adults [24]. The odors which have to be discriminated are relatively similar, since they only differ by the quantity of their components, rendering the DODT a relatively difficult test. It thus appears superior to other tests of olfactory discrimination (and identification), which are prone to ceiling effects in the general population [45]. In general, olfactory discrimination is an exceptionally valid measure of olfactory performance since it taps what is probably the most basic feature of the olfactory system [46,47].

The gender differences evident within the current results relate to others which show that especially in women, social integration and attachment are correlated to olfactory skills [2,22]. While the current sample sizes admittedly are small, the resulting power was sufficient to detect the large correlation in women. The difference between the correlation of olfactory abilities and mentalizing in women vs. men, together with the almost zero-correlation in men strongly indicate that there is no significant effect in men that can be detected, even with higher statistical power. With additional reference to the overall female superiority in empathic capacities on one hand [19], and olfactory performance on the other hand [18], it is suggested that women, compared to men, benefit from a higher integration of socio-olfactory functions. The findings of women responding more sensitively to social odors than men, both on the neural [48,49] and the behavioral level [50,51], support this suggestion.

Taken together, the current results suggest an intimate link between olfaction, which is among the phylogenetically oldest sense in vertebrates, and empathic skills, which are necessary for successful social interaction. Drawing on the discussion of “higher olfactory functions”, having been engaged in the evolution of social behavior in modern humans [43], the current study highlights yet another important indication of olfaction serving functions above and beyond smell [52], and suggests that the olfactory sense might be considered a social sense by nature.

## Figures and Tables

**Table 1:** Women’s and men’s performance, ratings, and sex hormone levels.

	Women	Men
	Md (+/− IQR)	Md (+/− IQR)
Olfactory discrimination (DODT)	8.00 (4.00)	7.50 (5.00)
Olfactory identification (BSIT)	11.00 (1.00)	11.00 (2.00)
Mentalizing (RMET)	26.00 (5.00)	25.00 (5.00)
Loneliness (UCLA loneliness scale)	1.80 (0.90)	1.70 (0.65)
State happiness	8.00 (2.00)	6.00 (3.00)
Estradiol (pg/mL)	4.84 (1.86)	3.40 (1.26)
Testosterone (pg/mL)	14.45 (10.81)	75.82 (46.74)

Notes. BSIT = “Brief Smell Identification Test”, DODT = “Düsseldorf Odor Discrimination Test”, RMET = “Reading the Mind in the Eyes” test, Md = Median, IQR = Interquartile Range.

**Table 2:** Correlations of mentalizing, loneliness, happiness and sex hormone levels with olfactory performance in women.

	DODT		BSIT	
	rho	*p* (uncorr. *p*)	rho	*p* (uncorr. *p*)
Mentalizing (RMET)	0.572 **	0.042 (0.007)	−0.002	>0.999 (0.993)
Loneliness (UCLA loneliness scale)	0.047	>0.999 (0.841)	0.385	0.564 (0.094)
State happiness	0.503 *	0.120 (0.020)	−0.001	>0.999 (0.996)
Estradiol (pg/mL)	0.209	>0.999 (0.376)	0.244	>0.999 (0.300)
Testosterone (pg/mL)	0.252	>0.999 (0.285)	0.211	>0.999 (0.371)

Notes. BSIT = “Brief Smell Identification Test”, DODT = “Düsseldorf Odor Discrimination Test”, RMET = “Reading the Mind in the Eyes” test, ** = Bonferroni-corrected *p* < 0.05, * = uncorrected *p* < 0.05.

**Table 3:** Correlations of mentalizing, loneliness, happiness and sex hormone levels with olfactory performance in men.

	DODT		BSIT	
	rho	*p* (uncorr. *p*)	rho	*p* (uncorr. *p*)
Mentalizing (RMET)	−0.117	>0.999 (0.623)	−0.367	0.672 (0.112)
Loneliness (UCLA loneliness scale)	−0.199	>0.999 (0.400)	−0.308	>0.999 (0.187)
State happiness	−0.390	0.534 (0.089)	−0.230	>0.999 (0.328)
Estradiol (pg/mL)	0.085	>0.999 (0.729)	−0.106	>0.999 (0.665)
Testosterone (pg/mL)	0.349	0.858 (0.143)	−0.163	>0.999 (0.505)

Notes. BSIT = “Brief Smell Identification Test”, DODT = “Düsseldorf Odor Discrimination Test”, RMET = “Reading the Mind in the Eyes” test.

## Data Availability

The datasets generated for this study are available upon request from the corresponding author.
